# Mechanisms and functional implications of ZDHHC5 in cellular physiology and disease

**DOI:** 10.1016/j.jlr.2025.100793

**Published:** 2025-04-01

**Authors:** Huicong Liu, Shuo Wen, Chang Xu, Xiaohong Kang, Eryan Kong

**Affiliations:** 1 The First Affiliated Hospital of Xinxiang Medical University, Xinxiang Medical University, Xinxiang, China; 2 Institute of Psychiatry and Neuroscience, Xinxiang Key Laboratory of Protein Palmitoylation and Major Human Diseases, Xinxiang Medical University, Xinxiang, China

**Keywords:** cancer, cardiac function, neurodegenerative diseases, substrate recognition

## Abstract

Post-translational lipid modification by palmitoylation is a reversible process crucial for maintaining cellular functionality. The palmitoyl acyltransferase zinc finger Asp-His-His-Cys motif-containing 5 (ZDHHC5) has garnered significant attention due to its roles in neurodegenerative diseases, oncogenesis, and cardiac function. ZDHHC5 recognizes substrates through diverse mechanisms and its activity is regulated by multiple factors. Highly expressed in the brain, liver, and heart, ZDHHC5 exerts regulatory functions in various cellular processes through self-regulation and substrate palmitoylation. This review summarizes ZDHHC5's regulatory roles in the nervous system, lipid metabolism and oncogenesis, highlighting its potential as a therapeutic target for neurological, lipid metabolic diseases, and cancer due to its involvement in diverse cellular processes and disease-associated dysfunctions.

Protein function regulation is crucial for cellular homeostasis, with post-translational modifications (PTMs) playing a key role in modulating protein activity, localization, and interactions (1, 2, 3). Among the numberous PTMs, S-palmitoylation—the attachment of palmitic acid to cysteine residues—has emerged as a critical modulator of various biological processes (4). This reversible modification, discovered over 40 years ago, regulates protein-membrane interactions, stability, and signaling, influencing a wide array of cellular functions (5). One of the key enzymes responsible for palmitoylation is ZDHHC5, a membrane of the palmitoyl acyltransferase family. ZDHHC5 has garnered significant attention due to its tissue-specific expression and its involvement in both normal physiological processes and disease mechanisms.

Recent studies have highlighted ZDHHC5’s diverse roles in the nervous system, tumor biology, and cardiac function. In the brain, ZDHHC5 is involved in synaptic plasticity, neuronal development, and circadian rhythm regulation, with dysregulation linked to neurodegenerative diseases such as Alzheimer’s disease (AD). In cancer, ZDHHC5 palmitoylates oncogenic proteins, contributing to tumor progression and highlighting its relevance for cancer therapies. The enzyme also regulates critical proteins in cardiac physiology, influencing heart contraction and ion transport, making it a significant player in cardiovascular diseases, including heart failure and arrhythmias. These findings underscore ZDHHC5’s pivotal role in both cellular homeostasis and disease pathophysiology.

Despite these advances, there remain critical gaps in understanding ZDHHC5’s regulatory mechanisms, substrate specificity, and broader functional implications. A comprehensive review is essential to consolidate the current knowledge and identify critical research directions. Exploring the interactions between ZDHHC5 and other PTMs, such as phosphorylation, adds another layer of complexity, as these modifications collaborate in regulating cellular processes and disease progression. Understanding these interactions will provide deeper insights into cellular signaling networks and may reveal new therapeutic strategies targeting ZDHHC5.

As research on ZDHHC5 continues to evolve, its significance in cell biology grows, offering potential therapeutic avenues for neurological, metabolic, and cardiac disorders. By elucidating the molecular mechanisms underlying ZDHHC5’s functions, this review aims to contribute to a broader understanding of its role in cellular physiology and its potential in bridging basic scientific research with clinical applications.

## Regulation of ZDHHC5 Enzyme Activity

ZDHHC5 has emerged as a pivotal enzyme in the realm of protein S-acylation, with its regulatory mechanisms increasingly elucidated over the past decade. The intricate interplay of post-translational modifications governing ZDHHC5’s enzymatic activity has been a focal point of recent research, revealing a complex network of regulatory pathways that modulate its function.

### Post-translational modifications

**Fig. 1:**
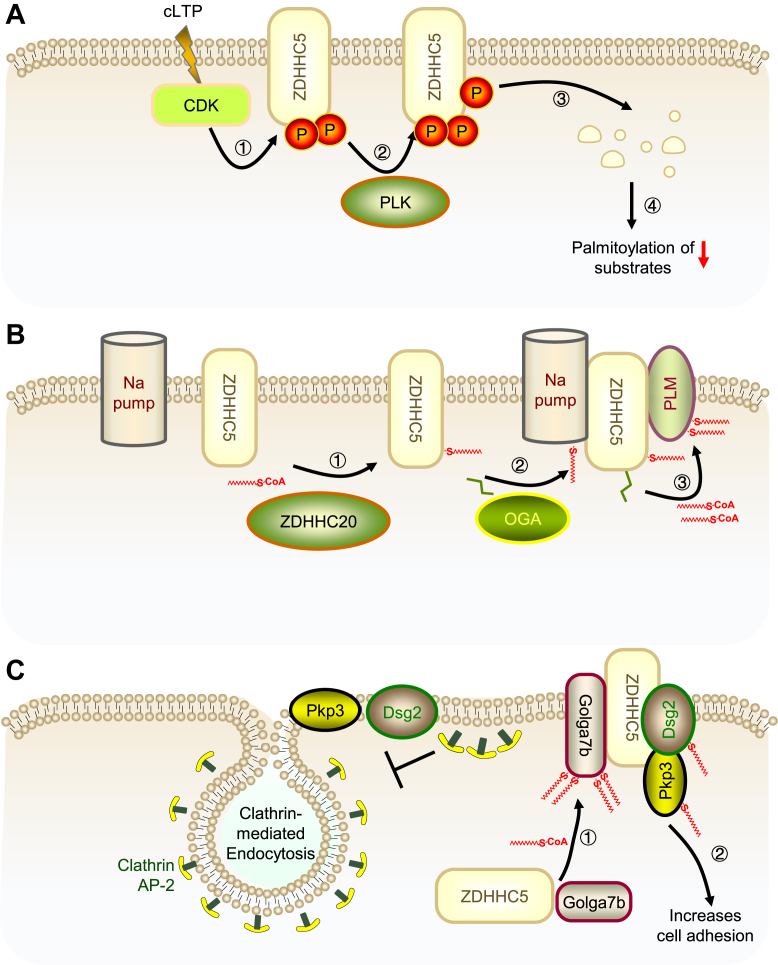
Molecular Mechanism Diagram of ZDHHC5 Enzyme Activity Regulation. A: CDK-PLK Signaling Axis Regulation of ZDHHC5 Activity. Step1, CDK5 as the priming kinase that phosphorylates serine–threonine–proline (STP) motifs in ZDHHC5 and enhances the degradation signal. Step2, PLK2 phosphorylates ZDHHC5, which is crucial for its proteasomal degradation. B: Palmitoylation by ZDHHC20. Step1, ZDHHC20 palmitoylates cysteine residues C236 and C237 in ZDHHC5, enhancing its interaction with the Na pump. This interaction further promotes the palmitoylation of PLM catalyzed by ZDHHC5. C: Stabilization by Golga7b. Golga7b acts as a chaperone protein, stabilizing ZDHHC5 on the cell membrane (Step1). Step2: This stabilization enhances the palmitoylation of substrate proteins Dsg2 and Pkp3, promoting their functional modification.

In the realm of enzymatic activity mechanisms, ZDHHC5 has emerged as a pivotal enzyme with its regulatory nuances being increasingly elucidated. The past decade has witnessed a substantial advancement in comprehending the intricate interplay of PTMs that govern the S-acylation activity of ZDHHC5. Notably, synaptic activity-induced phosphorylation events, mediated by cyclin-dependent kinase 5 (CDK5) and polo-like kinase 2 (PLK2), have been shown to target ZDHHC5 at specific serine and threonine residues (Ser569, Ser573, and Thr574), subsequently promoting its proteasomal degradation (Fig. 1A) (6). The carboxy-terminal PaCCT motif of ZDHHC5 is a critical determinant for its function, undergoing palmitoylation by another ZDHHC enzyme, ZDHHC20. This modification is instrumental in facilitating the interaction between ZDHHC5 and the Na^+^/K^+^ ATPase pump (Na^+^ pump), thereby orchestrating the recruitment of downstream substrates for palmitoylation (Fig. 1B) (7). Furthermore, the O-GlcNAcylation of ZDHHC5 at the S241 residue has been implicated in the enhanced recruitment of phospholemman (PLM) via the Na^+^ pump, underscoring the significance of this modification in the regulation of ZDHHC5's substrate interaction (8). Beyond the realm of PTMs, the activity of ZDHHC5 is also modulated by the availability of its substrate, palmitoyl-CoA, which is provided by acyl-CoA synthetases (ACSLs) (9).

### Chaperone proteins and subcellular localization

Chaperone proteins have been identified as crucial regulators of ZDHHC5 activity. Specifically, the palmitoylation of Golga7b by ZDHHC5 prevents the internalization of ZDHHC5 via clathrin-mediated endocytosis, thereby ensuring the stable positioning of ZDHHC5 on the cytoplasmic membrane (10). The co-localization of ZDHHC5 with Golga7b is essential for the proper positioning of desmosomal proteins, such as desmoglein-2 (Dsg-2) and plakophilin-3 (Pkp3), which are vital for the assembly and function of desmosomes (Fig. 1C) (11, 12, 13).

Collectively, these findings underscore the multifaceted regulatory mechanisms that govern ZDHHC5 function. From PTMs such as phosphorylation, palmitoylation, and O-GlcNAcylation to the involvement of chaperone proteins and substrate availability, ZDHHC5’s activity is tightly regulated at multiple levels. These insights not only enhance our understanding of protein S-acylation but also shed light on the broader role of ZDHHC5 in cellular processes. Further exploration of these regulatory pathways may provide new avenues for targeting ZDHHC5 in therapeutic contexts.

## Patterns of Substrate Recognition by ZDHHC5

Within the cellular proteome, ZDHHC5 is distinguished by its relatively low abundance, with estimated copy numbers ranging from approximately 1,500 to 60,000 in HeLa cells. Despite its scarcity, ZDHHC5 exhibits highly efficient enzymatic activity, enabling it to effectively modulate the functionality of its substrates (14). ZDHHC5, which harbors a PDZ-binding motif, belongs to the same subfamily as ZDHHC8. Its catalytic mechanism involves two key steps: initial engagement with palmitoyl-CoA, followed by the transfer of the palmitoyl moiety to the cysteine residue of the target protein (15, 16).

### Substrate recognition mechanisms

ZDHHC5’s substrate recognition is categorized into two distinct pathways: PDZ-dependent and non-PDZ-dependent. PDZ-dependent recognition is exemplified by substrates such as PSD95 (17), while non-PDZ-dependent recognition includes substrates like PLM (18). This diversity in substrate recognition is likely attributable to ZDHHC5’s unique C-terminal domain, which spans approximately 120 amino acids and plays a critical role in engaging non-PDZ-dependent substrates (17, 18).

Other ZDHHC proteins with PDZ-binding domains, such as ZDHHC17, ZDHHC8, and ZDHHC14, also exhibit substrate recognition mechanisms. Among these, ZDHHC17 demonstrates the most pronounced reliance on the PDZ domain for substrate binding (11, 19, 20). In contrast, ZDHHC5 exhibits minimal dependence on its PDZ-binding motif for substrate recognition, underscoring the considerable diversity in substrate specificity among palmitoyl acyltransferases (18).

### Dynamic subcellular localization

As a transmembrane protein with four transmembrane domains, ZDHHC5 exhibits dynamic subcellular localization, which is pivotal for its substrate recognition and regulatory functions (18, 21, 22). This dynamic distribution allows ZDHHC5 to precisely control and modulate its interactions with various substrates in a context-dependent manner.

Neuronal activity significantly influences the localization and function of ZDHHC5. Enhanced neuronal activity disrupts the endocytic trafficking of the ZDHHC5/PSD-95/Fyn complex, leading to the redistribution of ZDHHC5 from the synaptic membrane to the axon. In the axon, ZDHHC5 interacts with δ-catenin, augmenting its palmitoylation levels (17, 23, 24). These palmitoylated proteins are subsequently recycled to endosomes and trafficked back to synaptic sites, a process essential for the postsynaptic localization of δ-catenin and the stabilization of AMPA receptors. This dynamic localization of ZDHHC5 is critical for dendritic spine formation and the regulation of gene transcription, processes that are fundamental to learning and memory. By modulating the palmitoylation of key synaptic proteins, ZDHHC5 plays a central role in synaptic plasticity and neuronal function.

ZDHHC5’s substrate recognition mechanisms and dynamic subcellular localization highlight its versatility and efficiency as a palmitoyl acyltransferase. The ability of ZDHHC5 to engage with both PDZ-dependent and non-PDZ-dependent substrates, coupled with its activity-dependent localization, underscores its critical role in regulating synaptic function and neuronal plasticity. Further research into these mechanisms will provide deeper insights into the molecular basis of ZDHHC5’s functions and its potential as a therapeutic target in neurological disorders.

## Regulatory role of ZDHHC5 in fatty acid metabolism

ZDHHC5 is a critical regulator of fatty acid metabolism, influencing key processes such as fatty acid uptake, distribution, oxidation, and storage. Further research into the molecular mechanisms underlying ZDHHC5's functions in fatty acid metabolism will not only deepen our understanding of metabolic regulation but also open new avenues for therapeutic interventions in metabolic and neurodegenerative diseases. Below is a detailed description of the specific regulatory functions of ZDHHC5 in the fatty acid metabolism process.

### Regulation of CD36 and fatty acid uptake

**Fig. 2:**
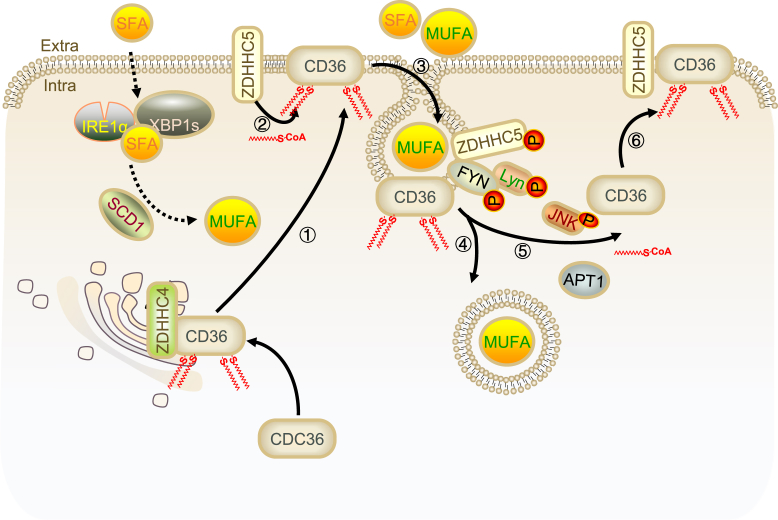
ZDHHC5 Mediates the Palmitoylation of CD36 to Regulate Fatty Acid Uptake. Step1, ZDHHC4, another palmitoyl acyltransferase, operates in the endoplasmic reticulum to catalyze CD36 palmitoylation necessary for protein folding. Step2, ZDHHC5 catalyzes the anchoring of CD36 to the cell membrane, essential for its membrane localization and fatty acid uptake activity. Step3, CD36 binds to fatty acids (FA) and facilitates their internalization through vesicle-dependent mechanisms. Step4, the palmitoylation of CD36 is associated with flotillin-dependent endocytosis, which can be enhanced by USMB treatment. Step5, APT1 mediates the depalmitoylation of CD36, leading to its detachment from the cell membrane. Step6, ZDHHC5 catalyzes the re-anchoring of CD36 to the cell membrane.

The translocase CD36, a key facilitator of fatty acid uptake, is dynamically regulated by ZDHHC5 and the depalmitoylation enzyme APT1. This regulation is critical for CD36-mediated fatty acid internalization. Upon binding fatty acids, CD36 undergoes APT1-mediated depalmitoylation, leading to its detachment from the cell membrane and the activation of downstream mediators such as LYN, which facilitates the internal transport of lipid cargoes. Subsequently, ZDHHC5 catalyzes the re-anchoring of CD36 to the cell membrane, a process essential for CD36’s membrane localization and fatty acid uptake activity (21, 25, 26). While ZDHHC5 is primarily localized to the cell membrane, consistent with its role in fatty acid uptake, ZDHHC4, another palmitoyl acyltransferase, operates in the endoplasmic reticulum, where it catalyzes CD36 palmitoylation necessary for protein folding (Fig. 2) (21). The palmitoylation of CD36 by ZDHHC5 is associated with flotillin-dependent endocytosis, a process that can be enhanced by ultrasound microbubble (USMB) treatment (27).

The molecular mechanism by which ZDHHC5 and APT1 coordinate CD36 dynamic palmitoylation and de-palmitoylation is basically clear. However, the role of other potential regulatory factors (kinases) in this process is largely unknown. Future studies should aim to identify these factors and explore how they influence ZDHHC5-CD36-mediated fatty acid uptake. Additionally, the potential therapeutic applications of modulating CD36 palmitoylation in metabolic diseases warrant further investigation.

### Role in fatty acid oxidation and AD

Long-chain fatty acyl-CoA synthetases (ACSLs), rate-limiting enzymes in fatty acid oxidation (FAO), convert palmitic acid to palmitoyl-CoA, which drives ZDHHC5-mediated palmitoylation of Cys194 in CLOCK, thereby regulating tumorigenesis (9, 28, 29). This establishes ZDHHC5's involvement in FAO.

ZDHHC5 is highly expressed in microglia and is implicated in the pathogenesis of AD. In AD brains, particularly those with the APOE4/4 genotype, ACSL1-positive microglia exhibit a strong correlation with ZDHHC5 activity. Fibrous Aβ (fAβ)-induced expression of ACSL1, triglyceride synthesis, and lipid droplet accumulation in induced pluripotent stem cell-derived microglia (iMG) with the APOE4/4 genotype lead to tau protein phosphorylation and neurotoxicity, processes dependent on APOE (30). Thus, ZDHHC5 plays a critical role in AD by modulating lipid metabolism, lipid droplet accumulation, and neurotoxicity in iMG cells. These findings provide novel insights into the molecular mechanisms underlying AD and highlight ZDHHC5 as a potential therapeutic target for this condition.

Despite these insights, several critical questions remain unanswered. For example, the detailed molecular mechanisms by which ACSL1 regulates ZDHHC5 activity and lipid droplet accumulation in microglia are not fully understood. Additionally, the role of ZDHHC5 in the cross-talk between lipid metabolism and neuroinflammation in AD pathogenesis is still unclear. Future research should focus on elucidating these mechanisms and exploring the potential of targeting ZDHHC5 to modulate lipid metabolism and reduce neurotoxicity in AD.

### Regulatory role of ZDHHC5 in the central nervous system

Palmitoylation plays a pivotal role in regulating a wide array of biological processes within the central nervous system (CNS). These processes encompass neuronal maturation, synaptic plasticity, neuroinflammation, and cognitive functions such as learning and memory (31, 32, 33). Among the palmitoyl acyltransferases, ZDHHC5 has emerged as a key regulator, exhibiting robust expression in the brain and contributing significantly to the development and function of the nervous system (9, 22).

#### Neuronal differentiation and development

**Fig. 3:**
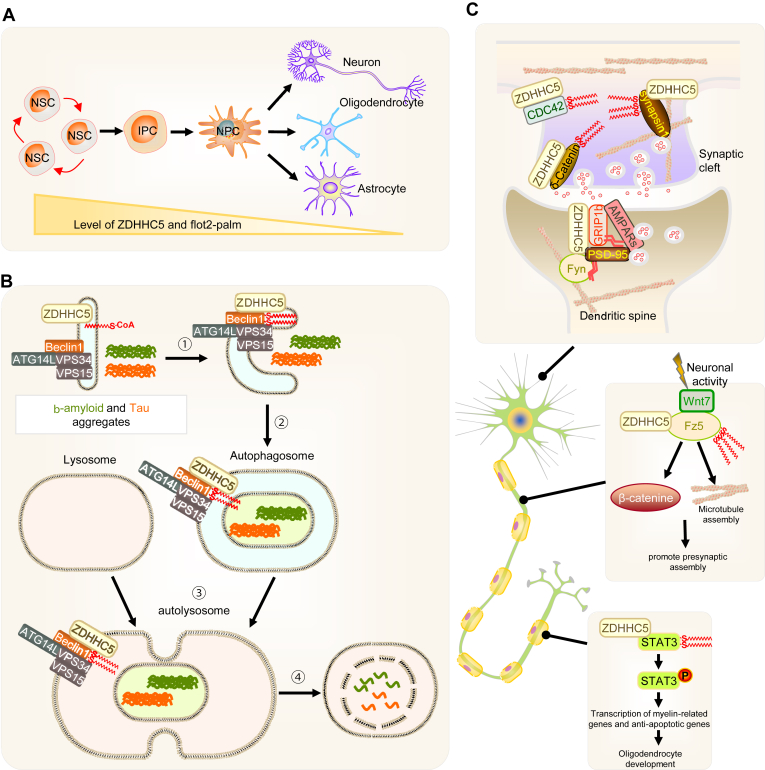
Regulatory function of ZDHHC5 in the central nervous system. A: During neuronal differentiation, ZDHHC5 undergoes targeted degradation, inhibiting the palmitoylation of FLOT2. B: Beclin1 palmitoylation and autophagic process. ZDHHC5 mediates the palmitoylation of Beclin1(Step1), promoting the formation of autophagosomes (Step2) and autolysosome (Step3). This process maintains protein homeostasis and alleviates the accumulation of β-amyloid and Tau aggregates, which is critical for mitigating the progression of AD. C: ZDHHC5 regulates neuronal development and synaptic plasticity by regulating palmitoylation modification levels of CDC42, δ-Catenin, synapsin1, GRIP1b, PSD95, and Fz5 in neurons (upper). ZDHHC5 catalyzes the S-acylation of the three C-terminal cysteine residues of Fz5, thereby regulating its localization at the cell surface and along axons, enhancing its stability, and reducing endocytosis (middle). ZDHHC5 activates STAT3 via palmitoylation, further activation of STAT3 phosphorylation, and promoting the transcription of genes critical for myelination (bottom).

During neuronal differentiation—a critical phase in nervous system maturation—ZDHHC5 undergoes targeted degradation. This degradation inhibits the palmitoylation of flotillin-2 (FLOT2), positioning ZDHHC5 as a crucial modulator of both FLOT2 modification and the broader process of neural stem cell differentiation (34). The regulation of ZDHHC5 expression and activity thus represents an essential mechanism for controlling neural stem cell differentiation, underscoring the importance of understanding the interplay between ZDHHC5 and FLOT2 in neuronal development (Fig. 3A).

However, the specific molecular mechanisms underlying ZDHHC5-mediated degradation remain to be fully elucidated. Future studies should focus on identifying the signaling pathways and regulatory factors involved in this process. Additionally, the role of ZDHHC5 in the expression of other types of neural cells, such as microglia and astrocytes, remains largely unexplored. Investigating these aspects could provide deeper insights into the multifaceted role of ZDHHC5 in CNS development.

#### Autophagy and neurodegenerative diseases

Autophagy, a cellular process that mitigates the accumulation of toxic protein aggregates, plays a vital role in the pathogenesis of neurodegenerative diseases (35, 36, 37). Notably, autophagy levels decline with age, a phenomenon paralleled by a similar age-related reduction in ZDHHC5 expression (38, 39). Downregulation of ZDHHC5 leads to decreased palmitoylation of Beclin1, impairing the assembly of the PI3KC3-C1 complex and disrupting the autophagic process. This disruption is associated with increased phosphorylation and pathological accumulation of Tau in neurons, as well as elevated Aβ deposition in 5×FAD mice, a model of AD (Fig. 3B). Furthermore, ZDHHC5 deficiency in AD model mice results in excessive microglial activation, potentially exacerbating neuroinflammation (40). However, the role of ZDHHC5 in the cross-talk between autophagy and neuroinflammation in neurodegenerative diseases is still unclear. Future research should focus on elucidating these mechanisms and exploring the potential of targeting ZDHHC5 to modulate autophagy and reduce neuroinflammation in conditions such as AD.

Additionally, elevated levels of PKCδ in hypothalamic microglial cells of patients with hyperlipidemia highlight the ZDHHC5-PKCδ pathway as a potential therapeutic target for hepatic steatosis (41). These findings collectively suggest that ZDHHC5 plays a critical role in maintaining autophagy and mitigating the progression of neurodegenerative diseases.

Additionally, the development of specific inhibitors or activators of ZDHHC5 that can modulate its activity in neurons and microglia could provide new therapeutic strategies for neurodegenerative diseases. Evaluating the efficacy and safety of these compounds in preclinical models of AD will be essential for their potential translation to clinical applications.

#### Synaptic plasticity and cognitive function

ZDHHC5 is localized to the cell membrane, where it colocalizes with the small GTPase Cdc42. This interaction is essential for the palmitoylation of Cdc42, a modification that regulates Cdc42-mediated gene transcription and the formation of dendritic spines in hippocampal neurons, processes vital for synaptic plasticity and memory formation (42). ZDHHC5 also interacts with δ-catenin, catalyzing its palmitoylation, which is necessary for the proper localization of δ-catenin at postsynaptic membranes and the stabilization of AMPA receptors. These processes are instrumental in learning and memory in murine models.

A reciprocal relationship exists between palmitoylation and phosphorylation in the regulation of ZDHHC5 target proteins. For instance, ZDHHC5 regulates the synapsin1-F-actin interaction through the palmitoylation of synapsin1. Notably, increased phosphorylation of synapsin1 reduces its palmitoylation, impacting synaptic vesicle clustering and release dynamics (43). Additionally, ZDHHC5 modifies the Frizzled receptor (Fz5) via palmitoylation, influencing its surface distribution along axons in response to neuronal activity. Impaired palmitoylation of Fz5 disrupts its assembly at presynaptic sites, further implicating ZDHHC5 in synaptic function (Fig. 3C middle) (44).

ZDHHC5 exhibits activity-dependent subcellular localization, with neuronal activity influencing its membrane localization and functionality at postsynaptic sites. In the hippocampus, ZDHHC5 interacts with the PDZ3 domain of the scaffolding protein PSD95, mediated by its C-terminal domain. While this interaction does not appear to affect the palmitoylation status of PSD95 (17), it plays a role in stabilizing synaptic proteins. Under basal conditions, ZDHHC5 interacts with PSD95 and Fyn kinase, stabilizing these proteins at synaptic membranes through Fyn-mediated phosphorylation. Increased neuronal activity disrupts the ZDHHC5/PSD95/Fyn complex, leading to the internalization of ZDHHC5 and its association with δ-catenin in axons (23, 27). Additionally, while the interaction between ZDHHC5 and PSD95 has been shown to stabilize synaptic proteins, the functional consequences of this interaction on synaptic strength and plasticity remain unclear.

ZDHHC5 also interacts with GRIP1b through its C-terminal PDZ domain, a process critical for the palmitoylation of GRIP1b. This modification facilitates the subcellular localization of GRIP1b to dendritic transport vesicles, influencing AMPA receptor trafficking and synaptic plasticity (45, 46). The rapid turnover of ZDHHC5-mediated palmitoylation on GRIP1b underscores its role in the cycling of AMPA receptors and the dynamics of neuronal protein transport (Fig. 3C upper).

Recent studies have highlighted the role of synaptic activity in modulating ZDHHC5 function through palmitoylation and phosphorylation. Under conditions of chemical long-term potentiation (cLTP), ZDHHC5 protein levels decrease while its palmitoylation increases, suggesting that synaptic activity directly influences its stability and function. Enhanced phosphorylation of specific serine/threonine residues within the polo-box domain of ZDHHC5 following cLTP, further underscores the role of phosphorylation in its degradation (23). These findings emphasize the intricate regulatory mechanisms governing ZDHHC5’s role in synaptic function.

#### Oligodendrocyte development and myelination

Beyond its role in synaptic plasticity, ZDHHC5 is critical for oligodendrocyte development, which is essential for myelin formation in the CNS. Myelination is crucial for maintaining neuronal integrity and efficient signal conduction (47, 48). ZDHHC5 contributes to oligodendrocyte development by activating STAT3 via palmitoylation. Reduced ZDHHC5 activity impairs STAT3 phosphorylation and activation, compromising the transcription of genes critical for myelination and anti-apoptotic pathways. This inhibition hinders oligodendrocyte maturation and myelin synthesis (Fig. 3C botom) (49). These findings suggest that targeting ZDHHC5 could represent a promising therapeutic strategy for demyelinating disorders, offering the potential for enhancing oligodendrocyte regeneration and promoting myelin repair.

ZDHHC5 plays a crucial role in the nervous system by regulating the palmitoylation of various substrates, a function that has been extensively studied. However, several key aspects remain unclear. First, the dynamic regulation of ZDHHC5 through phosphorylation and its effects on protein stability and function during different forms of synaptic plasticity, such as LTP and LTD, need further clarification. Identifying the specific kinases and phosphatases that modulate ZDHHC5 phosphorylation under varying activity conditions could provide deeper insights into its regulatory mechanisms. Future research should focus on unraveling these complex regulatory networks and identifying new substrates to comprehensively understand ZDHHC5’s roles in synaptic plasticity, cognitive function, and neuroinflammation. Addressing these research directions will enhance our understanding of ZDHHC5’s multifaceted role in the nervous system and its potential for therapeutic applications.

### Regulatory role of ZDHHC5 in oncogenesis

Palmitoylation is a dynamic and reversible modification that rapidly alters the membrane affinity of target proteins, impacting their localization, accumulation, secretion, stability, and function. These changes are directly correlated with cancer cell growth, survival, and therapeutic resistance (50, 51). ZDHHC5 has attracted considerable attention due to its elevated expression in tumor tissues, where it regulates key oncogenes and tumor suppressors, thus facilitating tumorigenesis and progression in various cancers, including gliomas, breast cancer, and pancreatic cancer (52, 53, 54). Understanding how ZDHHC5 modulates the function of substrates in cancer cells is therefore crucial for the development of targeted therapies. However, the potential of ZDHHC5 as a prognostic biomarker for various cancers needs to be validated.

**Fig. 4:**
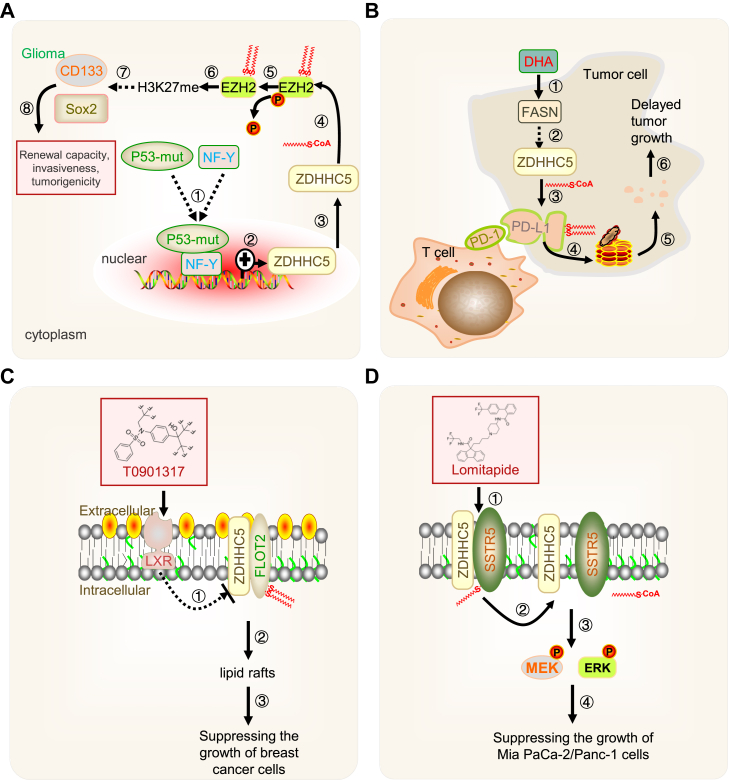
Regulatory function of ZDHHC5 in the tumor cells. A: Role in glioma. Glioma cells with p53 mutations often exhibit elevated ZDHHC5 levels due to cooperative effects between mutated p53 and the nuclear transcription factor NF-Y (step1-3). ZDHHC5 palmitoylates EZH2 (Step4), reducing its phosphorylation (Step5) and decreasing H3K27me3 levels (Step6). This epigenetic modification contributes to the suppression of tumor suppressor genes, promoting glioma cell proliferation and tumorigenesis (Steps 7 and 8). B: DHA inhibition. DHA reduces FASN expression in cancer cells (step1), inhibiting ZDHHC5 activity (Step2) and promoting PD-L1 degradation via the ubiquitin-proteasome pathway (Step3). C: LXR Agonist T0901317. This compound modulates ZDHHC5 expression and regulates FLOT2 palmitoylation (Steps 1 and 2), exerting anti-proliferative effects in breast cancer cell models. D: Lomitapide inhibits signaling pathways in pancreatic cancer cells. Lomipapide competitively inhibits the binding of ZDHHC5 to SSTR5 (Step1), thereby reducing the palmitoylation level of SSTR5 (Step2) which subsequently leads to a decrease in the phosphorylation MEK and ERK (Step3), and exerts anti-tumor effects (Step4).

Studies have shown that higher levels of ZDHHC5 correlate with decreased survival and unfavorable clinical outcomes in patients with glioma, suggesting its potential as a prognostic marker (54, 55, 56). Notably, glioma cells harboring p53 mutations often exhibit elevated ZDHHC5 protein levels, likely due to cooperative effects between mutated p53 and the nuclear transcription factor NF-Y, which may enhance ZDHHC5 expression. ZDHHC5 overexpression in gliomas is linked to the palmitoylation of EZH2, resulting in reduced EZH2 phosphorylation and lower H3K27me3 levels (Fig. 4A). This epigenetic modification could contribute to the suppression of tumor suppressor genes, thereby promoting glioma cell proliferation, migration, and tumorigenesis. In contrast, the knockdown of ZDHHC5 in human neural stem cells (hNSCs) reduces their tumorigenic potential in mouse models (34). Furthermore, the liver X receptor (LXR) has been shown to suppress breast cancer cell growth by modulating cholesterol homeostasis in cell membranes (57, 58). ZDHHC5-mediated palmitoylation of FLOT2 indirectly affects the integrity of lipid rafts, which are crucial for various cellular signaling pathways.

In addition to its role in tumor growth, ZDHHC5 also influences immune evasion in cancers. PD-1, an immune checkpoint receptor overexpressed in various cancers, negatively regulates T-cell function by binding to its ligand, PD-L1, aiding cancer cells in evading immune surveillance (59, 60). ZDHHC5 is a critical enzyme regulating PD-L1 stability, as it palmitoylates PD-L1, enhancing its stability and promoting PD-L1-mediated immune suppression (Fig. 4B). While ZDHHC5-mediated palmitoylation of PD-L1 has been linked to immune suppression, its influence on other immune checkpoint molecules and immune cell signaling pathways is unclear. Understanding how ZDHHC5 modulates the tumor-immune interface could provide insights into combination therapies involving immune checkpoint inhibitors and ZDHHC5-targeted agents.

Several compounds, such as docosahexaenoic acid (DHA), T0901317, and lomitapide, have been identified as potential inhibitors of ZDHHC5-mediated palmitoylation, offering a promising therapeutic approach (53, 61, 62). DHA, for example, significantly reduces fatty acid synthase (FASN) expression in cancer cells, inhibiting ZDHHC5 activity and promoting the degradation of PD-L1 via the CSN5-dependent ubiquitin-proteasome pathway (Fig. 4B) (62). This positions DHA as a potential immune enhancer for cancer therapy. Similarly, LXR agonist T0901317 modulates ZDHHC5 and FLOT2 expression, exerting anti-proliferative effects in breast cancer cell models (Fig. 4C) (61). Although 2-BP, a non-specific palmitoylation inhibitor, has been employed in preclinical studies to suppress tumor formation by targeting ZDHHC5 (63, 64, 65, 66), its broader applicability as a therapeutic agent remains limited due to its non-specific nature. Additionally, lomitapide has demonstrated the ability to inhibit signaling pathways in pancreatic cancer cells by reducing MEK and ERK protein phosphorylation, exerting significant anti-tumor effects in animal models. Lomitapide's mechanism involves competitive inhibition of ZDHHC5 binding to SSTR5, thereby reducing MEK and ERK phosphorylation (Fig. 4D) (53). These findings provide a solid theoretical foundation for developing therapeutic strategies targeting ZDHHC5, although further research is necessary to confirm their clinical efficacy and safety.

Developing highly specific inhibitors or small molecules that selectively target ZDHHC5 without affecting other palmitoyltransferases is essential for advancing clinical applications. Moreover, the pharmacokinetics, pharmacodynamics, and potential toxicities of these inhibitors in humans remain largely unexplored, necessitating comprehensive preclinical and clinical studies.

### Regulatory function of ZDHHC5 in the heart

In cardiac physiology, the Na^+^/K^+^ ATPase is recognized as the most abundant primary active transport protein on the cardiomyocyte membrane. Dysfunction of this protein results in an abnormal increase in intracellular sodium ion concentrations, which can lead to a range of cardiovascular diseases, including myocardial hypertrophy, diastolic dysfunction, arrhythmias, and heart failure. These conditions affect over 64 million people globally, presenting a significant public health challenge and becoming one of the leading causes of death, imposing a heavy burden on both socioeconomic and healthcare systems (67).

Various pathological changes in cardiac function are closely linked to alterations in PTMs of proteins, with palmitoylation being one of the most extensively studied modifications. The role of palmitoylation in cardiac disease remains largely unexplored. Among the palmitoylating enzymes, ZDHHC5 has been well characterized due to its distinct localization at the cell surface, making it a key candidate for further investigation in cardiac pathophysiology (68).

**Fig. 5:**
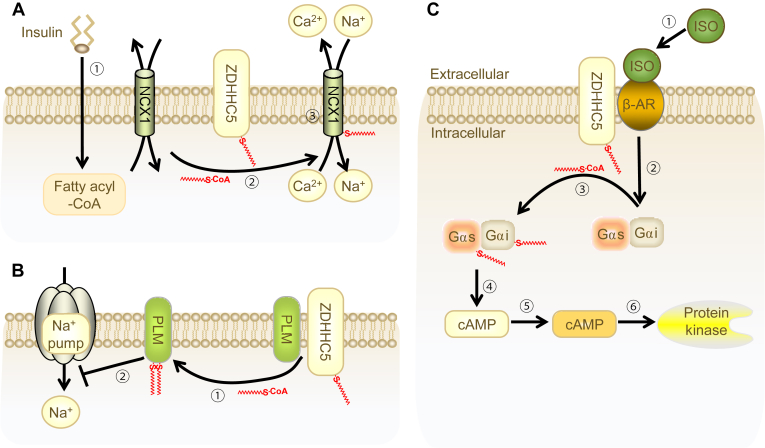
Regulatory function of ZDHHC5 in the heart. A: Insulin-Induced Palmitoylation. Insulin promotes synthesis of palmitoyl-CoA synthetase (step1). This process enhances the palmitoylation of the activity of ZDHHC5, promoting palmitoylation of NCX1 (step2) and impacting ion transport (step3). B: Palmitoylation of PLM. ZDHHC5 palmitoylates PLM (step1), which contributes to subsequent inhibition of the sodium pump (step2). C: β-Adrenergic Signaling. Step1, isoproterenol (ISO) Stimulation. ISO stimulates β-AR, enhancing the palmitoylation of Gαs and Gαi mediated by ZDHHC5. Step2, cAMP production. This process activates adenylate cyclase, producing the second messenger cAMP and activating downstream effectors such as PKA. Step3, cardiac contractile function. The palmitoylation of Gαs and Gαi is linked to the temporal regulation of cardiac contractile function.

Insulin plays a protective role in the heart by exerting anti-apoptotic, anti-inflammatory, and antioxidant effects. Studies have demonstrated that NCX1, a downstream target of insulin regulation, undergoes increased palmitoylation in response to insulin stimulation via an acyl-CoA synthetase-dependent mechanism. This, in turn, enhances the palmitoylation of the active site of ZDHHC5 (Fig. 5A) (69, 70, 71). Additionally, ZDHHC5 also palmitoylates NCX1 and PLM, both of which are involved in hypoxia/reperfusion injury (70). The increased phosphorylation of PLM contributes to its palmitoylation, thereby inhibiting the activity of the sodium pump (Fig. 5B). Although palmitoylation does not affect PLM's localization at the cell membrane, it reduces its half-life and regulates its turnover (8, 72, 73). However, the precise mechanisms by which ZDHHC5 expression and activity are regulated in response to cardiac stress, such as hypoxia/reperfusion injury, are not fully elucidated. Further studies are needed to identify the upstream signaling pathways and transcriptional regulators that control ZDHHC5 expression under these pathological conditions. Further studies are needed to identify the upstream signaling pathways and transcriptional regulators that control ZDHHC5 expression under these pathological conditions.

Beta-adrenergic receptors (β-ARs) are key regulators of cardiac contractile function, coupling with Gαs and Gαi proteins to activate or inhibit adenylate cyclase. This enzyme produces the second messenger cAMP, which activates multiple downstream effectors, including protein kinase A (PKA). Shortly after β-AR stimulation of cardiomyocytes, ZDHHC5, as a core regulator, exhibits increased palmitoylation levels and enhanced stability at the cell membrane. This simultaneously increases the palmitoylation levels of its substrates, Gαs and Gαi. The palmitoylation of Gαs and Gαi is linked to cAMP production and the temporal regulation of cardiac contractile function. In contrast, the knockdown of ZDHHC5 significantly impairs the regulation of this signaling pathway (Fig. 5C) (74).

In animal models of left ventricular hypertrophy (LVH) and heart failure (HF), as well as in human HF tissues, ZDHHC5 expression levels undergo significant changes. ZDHHC5 expression rapidly increases during the onset of LVH but decreases in the context of HF. However, paradoxically, palmitoylation of ZDHHC5 substrates, such as NCX1, is significantly reduced in LVH but increased in human HF. The palmitoylation of another substrate, PLM, remains unchanged across all conditions. Overexpression of ZDHHC5 in rabbit ventricular myocytes does not alter the palmitoylation levels of its substrates or the overall contractility of cardiomyocytes, suggesting that changes in ZDHHC5 expression may not be the primary driver of the pathological processes in these conditions (70). ZDHHC5 expression increases during LVH but decreases in HF, while the palmitoylation of its substrates shows opposing trends. The underlying reasons for this discrepancy and its functional implications for cardiac contractility and disease progression are unclear. Further research is needed to explore whether post-translational modifications of ZDHHC5 itself, such as phosphorylation or ubiquitination, or interactions with other regulatory proteins, contribute to these divergent outcomes. Understanding these mechanisms could reveal new theories for managing LVH and HF.

## Discussion

This review comprehensively examines the multifaceted roles of ZDHHC5 in cellular physiology and disease, highlighting its involvement in CNS, fatty acid metabolism, oncogenesis, and cardiac function. As a key palmitoyltransferase, ZDHHC5 regulates protein localization, stability, and function through S-palmitoylation, a reversible post-translational modification that influences a wide array of cellular processes. In this review, Table 1 summarizes the reported ZDHHC5 substrates, their physiological functions, and their modification sites. Despite significant progress in understanding ZDHHC5's functions, several critical gaps remain, particularly in its therapeutic potential and its role as a biomarker for disturbed energy metabolism in various diseases.

**Table 1:** Known substrates of ZDHHC5 and palmitoylation modification sites

Function	NO.	Substrates	Palmitoylation Sites	References
CNS	1	CDC42	Cys188,C189	Wirth A, *et al.* 2022 (42).
	2	PSD95	C3,C5	Zhang Y, *et al.* 2021 (75).
	3	GRIP1b	Unknown	Woodley KT, *et al.* 2019 (13).
	4	Fz5	C537,C538,C539	Teo S, *et al.* 2023 (44).
	5	Synapsin1	C223,C360,C370	Yan P, *et al.* 2022 (43).
	6	STAT3	C687,C712	Ma Y, *et al.* 2022 (49).
	7	δ-Catenin	C960 and C961	Brigidi GS, *et al.* 2014 (24).
Fatty acid metabolism	1	CD36	C3,C7,C464,C466	Tao N, *et al.* 1996 (76).
	2	PKCδ	Unknown	Wang YH, *et al.* 2024 (41).
	3	CLOCK	C194	Peng F, *et al.* 2024 (9).
Autophage	1	Beclin1	C137,C159	Guo R, *et al.* 2024 (40).
	2	Golga7b	C33,C78,C90,C141	Woodley KT, *et al.* 2019 (13).
	3	desmoglein-2	Unknown	Woodley KT, *et al.* 2019 (13).
	4	plakophilin-3	Unknown	Woodley KT, *et al.* 2019 (13).
Oncogenesis	1	EZH2	Cys571,Cys576	Chen X, *et al.* 2017 (54).
	2	PD-L1	C272	Yao H, *et al.* 2019 (64).
	3	FLOT2	C4,C20	Li Y, *et al.* 2012 (34).
	4	SSTR5	Cys134	Wang Y, *et al.* 2023 (53).
Heart	1	NCX1	C739	Reilly L, *et al.* 2015 (71).
	2	PLM	C40,C42	Tulloch LB, *et al.* 2011 (73).
	3	Gαs and Gαi	Unknown	Chen JJ, *et al.* 2020 (10).

While this review highlights ZDHHC5's involvement in neurodegenerative diseases, cancer, and cardiovascular disorders, it is true that direct evidence linking ZDHHC5 modulation to therapeutic outcomes is still limited. For instance, in AD, ZDHHC5 has been implicated in modulating lipid metabolism and neurotoxicity in microglia, but the exact mechanisms by which ZDHHC5 contributes to AD pathogenesis remain unclear. Future studies should focus on developing specific inhibitors or activators of ZDHHC5 and evaluating their efficacy in preclinical models of AD. Similarly, in cancer, ZDHHC5's role in palmitoylating oncogenic proteins like EZH2 and PD-1 suggests its potential as a therapeutic target.

Another critical area for future research is the development of specific ZDHHC5 inhibitors. The development of highly specific ZDHHC5 inhibitors, as opposed to non-specific palmitoylation inhibitors like 2-BP, is crucial for advancing clinical applications. The pharmacokinetics, pharmacodynamics, and potential toxicities of these inhibitors must also be thoroughly investigated in human trials. While compounds like DHA, T0901317, and lomitapide have shown promise in preclinical studies, their non-specific effects and potential off-target actions still needs further study. Developing highly specific inhibitors or small molecules that selectively target ZDHHC5 without affecting other palmitoyltransferases is essential for advancing clinical applications.

Indeed, ZDHHC5's involvement in lipid metabolism and fatty acid oxidation suggests that its dysregulation could be a downstream effect of metabolic disturbances. For example, in AD, lipid droplet accumulation in microglia is linked to lipid droplet accumulation and neurotoxicity, which are known to occur in the disease (77, 78). Similarly, in cancer, the shift from oxidative phosphorylation to fermentation metabolism is a hallmark of tumor cells (79, 80, 81). ZDHHC5's role in palmitoylating proteins involved in lipid metabolism could be a reflection of this metabolic reprogramming. Future research should explore whether ZDHHC5 levels or activity could serve as a biomarker for metabolic dysfunction in these diseases, potentially broadening its diagnostic and prognostic utility.

One area that requires further exploration is the substrate specificity of ZDHHC5. While several substrates have been identified, the full spectrum of ZDHHC5's targets remains poorly characterized, particularly in the context of disease. For example, in cancer, identifying additional ZDHHC5 substrates could reveal novel therapeutic targets and deepen our understanding of its oncogenic role. Additionally, the interplay between ZDHHC5-mediated palmitoylation and other post-translational modifications, such as phosphorylation and ubiquitination, is still poorly understood. Future studies should focus on unraveling these complex regulatory networks to gain a comprehensive understanding of ZDHHC5's role in cellular signaling and disease progression.

In conclusion, ZDHHC5 plays a critical role in a wide range of cellular processes and diseases, making it a promising target for therapeutic intervention. However, significant gaps remain in our understanding of its regulatory mechanisms, substrate specificity, and broader functional implications. Addressing these gaps will require a multidisciplinary approach, combining biochemistry, cell biology, genetics, and bioinformatics. By elucidating the molecular mechanisms underlying ZDHHC5's functions, we can pave the way for the development of novel therapeutic strategies to improve treatment outcomes and patient prognosis for a range of related diseases.

## Conflict of interest

The authors declare that they have no conflicts of interest with the contents of this article.
